# Development of a Website for Disseminating Knowledge About listening Effort for Professionals

**DOI:** 10.1055/s-0043-1777803

**Published:** 2024-03-15

**Authors:** Heloisa de Miranda Cantuaria Alves, Jerusa Roberta Massola de Oliveira, Sandra Lia do Amaral, Vitor Engrácia Valenti, Maria Fernanda Capoani Garcia Mondelli

**Affiliations:** 1 Postgraduate Program in Speech Pathology, Bauru School of Dentistry, University of São Paulo (USP), Bauru, SP, Brazil; 2 Hearing Health Divison, Hospital for Rehabilitation of Craniofacial Anomalies, University of São Paulo (USP), Bauru, SP, Brazil; 3 Departament of Physical Education, São Paulo State University (UNESP), Bauru, SP, Brazil; 4 Department of Speech Therapy, Faculty of Philosophy and Sciences, São Paulo State University (UNESP), Marília, SP, Brazil; 5 Department of Speech Therapy, Faculty of Dentistry of Bauru, University of São Paulo (USP), Bauru, SP, Brazil

**Keywords:** listening effort, health education, educational technology, audiology

## Abstract

**Introduction** Permanent education in health aims to ensure that professionals are constantly learning in the workplace and in the last few years institutions resorted to the technology-mediated education modality and new teaching possibilities were explored. In Brazil, between 2017 and 2021, only six articles and five monographs were published about listening effort.

**Objective** The objective of this study was to develop a website with scientific content on the topic listening effort for Speech -Language Therapist and Audiologist with free online access.

**Methods** The study was carried out in five stages: Analysis, contemplating the search for scientific materials to prepare the material. Design, in which the writing and design of the website was carried out. Development, carrying out the adequacy of the online material. Implementation, a stage in which professionals in the area evaluated the quality of the material after consenting to participation through a free and informed consent term. Review, stage in which the researcher analyzed the evaluators' responses.

**Results** The five stages of elaboration of the website were carried out, which was evaluated by professionals in the area. The average of responses to all applied questions rated the website as “superior”.

**Conclusion** The website development was validated for online availability.

## Introduction

During the corona virus disease (COVID-19) pandemic, education, even if temporarily, needed to be restructured at all levels, like most other social activities. Both basic education schools and postgraduate institutions resorted to the technology-mediated modality to give continuity to the teaching processes. Even with the of most of the population, new teaching possibilities were explored, and can be implemented in institutions that previously only offered face-to-face teaching. 1 Furthermore, there is a clear trend towards virtual scientific activities to strengthen the knowledge after the COVID-19 pandemic 2 3 4_._

Permanent education in health aims to ensure that professionals are constantly learning in the workplace, transforming their reality and that of the population they serve, ensuring comprehensive care. 5

In Brazil, a study found that professionals show interest indifferent modalities of postgraduate studies, and technology-mediated education provides the means to learn new knowledge. Remote teaching makes professional development possible even for those who work in regions with difficult access to face-to-face teaching, removing mobility and schedule limitations to exchange knowledge. 6

Bearing these factors in mind, we can see the importance of developing materials with a scientific theoretical framework so that education in the health sector is an accessible reality with quality teaching.

In Brazilian literature, the theme of Listening Effort is still rarely approached. Conducting a search in the Scielo-Brasil, Embase, and PubMed databases, using the corresponding Portuguese term “esforço auditivo”, 14 studies were found—of which 6 articles were published in journals and 8 were monographs, all published between 2017 and 2023. From that total, there were 2 monographs and 2 articles about creating materials for the application of behavioral assessments (please see supplemental file Appendix 1 ).

**Appendix 1:** Brazilian Materials on Listening Effort

Title (Author, Year of publication)	Objective	Conclusion
Listening effort and working memory capacity in the hearing impaired: an integrative literature review (Guijo et al., 2018) 24	- To review the literature on behavioral methods for assessing listening effort.	- The dual-task paradigm proved to be sensitive to measure listening effort.
Listening effort and fatigue in hearing impaired adolescents – use of the FM system (Cruz, 2018) 25	- To analyze listening effort and fatigue in adolescents with HL, users of hearing aids, with or without FM system. - To compare the performance of normal hearing adolescents with adolescents with HL in the dual-task paradigm. - To analyze impact of noise on learning. - To investigate the impact of using the FM system in the classroom.	- The Paleta platform was effective to verify the occurrence of listening effort and the FM system proved to be effective to reduce listening effort in the studied population. - Regarding fatigue, no significant differences were found between the groups. -The complaints of adolescents with HL significantly decrease with the use of the FM system. - Most teenagers using the FM system were satisfied and were making effective use of their devices in the classroom.
Physiological methods as indices for measuring listening effort: an integrative literature review (Guijo & Cardoso, 2018) 26	- To review the scientific literature on the objective assessment of listening effort in people without HL.	- Despite skin conductance presenting more accurate results, there is no consensus regarding the best objective method to measure listening effort.
Effort to hear and auditory aging: analysis of functional relationships between age, auditory sensitivity and divided attention (Santana, 2017) 27	- To verify the relationship of auditory sensitivity and divided attention with the effect of aging on listening effort.	- Age impacts listening effort. - Proportionally inverse relationship between divided attention and listening effort. - Interactions of influence between the variables of age, auditory sensitivity, divided attention and listening effort.
Content validation and response processes of an instrument for measuring listening effort (Guijo, 2019) 28	- To validate the content of an instrument for measuring listening effort.	- The instrument is suitable for its construction.
Measurement of listening effort using a Brazilian Portuguese dual-task paradigm: a pilot study (Guijo et al., 2019) 29	- To measure the occurrence of listening effort in normal listeners and analyze the clinical significance of the participants' performance.	- The proposed paradigm was sensitive to quantify the listening effort, making it possible to measure its occurrence in this population.
Content validation of an instrument to measure listening effort (Guijo et al., 2020) 30	- To validate listening effort assessment instrument in people with HL.	- The material is suitable for use to measure listening effort.
Analysis of listening effort in individuals with unilateral hearing loss adapted with the CROS system (Gução, 2020) 31	- To check listening effort in people with unilateral HL before and after using the CROS system. - To verify your restriction of participation and quality of life.	- The use of the CROS system was effective to eliminate participation restriction, improve quality of life and reduce listening effort.
Assessment of listening effort using amplification through a dual-task paradigm and pupillometry (Senis, 2020) 32	- To verify the performance of elderly users of hearing aids in the assessment of listening effort.	- It is necessary to improve the methods of assessment of listening effort in the elderly. - Pupillometry was not effective for this purpose in this study.
Listening effort and recording of parasympathetic control of the heart during sentence recognition: a pilot study (Guijo et al., 2020) 33	- To compare the heart rate variability in different listening situations and verify the sensitivity of this method to assess the listening effort.	- The recording of heart rate variability was not sensitive for the purpose of this study.
The effects of using hearing aids and a frequency modulated system on listening effort among adolescents with hearing loss. (Cruz et al., 2020) 34	- To compare the listening effort of adolescents with HL with hearing aids and using hearing aids and FM system and adolescents without hearing loss. -To develop website for secondary task application for Dual Task Paradigm.	- The FM system reduces listening effort in teenagers with hearing loss. - The site proved to be effective for the purpose.

**Abbreviations:** FM, frequency modulation; HL, hearing loss.

Through this narrative summary review of the literature, it is possible to observe the modernity and scarcity of the subject theme in the Brazilian literature. Thus, more works on the theme are necessary in order to update and inform professionals and people with hearing difficulties about this aggravating factor in quality of life. Therefore, the objective of this work was to develop a website with scientific content on the topic Listening Effort for speech–language therapists and audiologists with free online access.

## Methods

This is a study of elaboration and development of educational material in the format of a website based on scientific basis, developed according to the ethical standards established by the National Commission for Ethics in Research and sent for consideration by the Ethics Committee in Research on Human Beings of the proponent institution, respecting resolution 466/12. It was approved under protocol number 5.040.411.

The elaboration of the website was carried out in five stages, as proposed by the Analyze, Design, Develop, Implement, and Evaluate (ADDIE) model, which consists of the five stages that make up the acronym that names. 7

The five steps are described below:

## Step 1–Analysis

Stage of literature search on the topic Listening Effort, addressing the history of the studies, its definition, the cognitive process, the explanatory model, assessment, and intervention approaches. In addition, a search for materials on the more appropriate way to development of educational websites was carried out. The search for scientific materials was carried out in the SciELO, PubMed, LILACS–BIREME, EMBASE, Cochrane Library, CINAHL, and Web of Science databases, using the English descriptors: “listening effort”, “auditory fatigue” alone and in combination with the following descriptors: AND “evaluation” OR “measures”.

For greater scope of the search, time or type of publication was not delimited. Original articles, literature reviews, dissertations, and theses were considered.

## Step 2–Design

In step 2, the website's texts and design were elaborated, through the free Webnode platform that allows website development from a chosen model. For the development of adapted images and definition of the site layout, there was a collaboration with a professional designer from the Educational Technology sector of the Institution. This professional edited the images that made up the website and guided the choice of layout and arrangement of elements.

For ease of reading, the chosen design features high contrast between the background and font color, and the font has a simple stroke and standard size. The images used are authored by the research team or the online image repositories, and were appropriately cited, without the need for prior authorization by the repositories.

## Stage 3–Development

In step 3, during the adaptation of the material to the online platform, reviews were carried out by two reviewers participating in the research group who have expertise in the preparation of teaching materials in audiology. Both were audiologists and reviewed the website weekly. The website has the following access tabs: Home, Listening Effort, Understanding the Cognitive Process, Objective Assessment, Subjective Assessment, Behavioral Assessment, Therapeutic Resources, Testimonials, References, and Contact.

## Step 4–Implementation

At this stage, the evaluation material was disseminated through social media so that speech–language therapy and audiology professionals could evaluate the website content in order to guarantee the online material's reliability and quality In messaging applications, disclosure occurred to groups of speech–language therapists and audiologists, reaching more than 219 professionals only in the first sharing. The material was also disseminated on social networks to all users, through fixed and temporary posts. In the fixed post, there were 48 interactions with users. The temporary post, published on several profiles, had an average reach of 1,000 views. The sample was obtained for convenience. Participants who were not professionals in the field were excluded; those who had already evaluated the material previously were also excluded, avoiding duplicate responses.

To carry out the evaluation, each participant filled in the free and informed consent term, authorizing the use of their answers for the development of the material. To assess its quality, the Suitability Assessment of Materials for evaluation of health-related information for adults (SAM) in its version adapted to Brazilian Portuguese 8 were used.

For the application of the questionnaire, the Google Forms tool was used, with the free and informed consent term on its first page. After reading the term, the button to accept participation in the research and the link to access the website were made available.

To profile the website evaluators, the following pages in the form had questions about the participant's time and institution of graduation, and whether they had experience in the area of Audiology, then the SAM questions were made available.

The questionnaire was also made available to speech-language pathology and Audiology students, in order to obtain more comprehensive information about the material in relation to its usability and quality of information.

## Step 5–Assessment

During the fifth stage, the research team made the necessary modifications. The criterion used was the statistical analysis of the evaluators' answers in order to verify the consistency between their answers and the need to change the original material.

The SAM score is given by the sum of all the factors analyzed, with a maximum of 44 points; this was determined according to the recommendation of other authors. 8

The interpretation of the adequate score can be “superior” (70 to 100% of excellent/adequate answers), “adequate” (40 to 69% of excellent/adequate answers), or “not acceptable” (0 to 39% optimal/adequate answers) considering the sum of all questions from all categories. 8 9

For the evaluation of the “Esforço Auditivo” website, all SAM questions were consistent with the content and, therefore, the answer option “N/A” was not available. Therefore, a descriptive analysis was performed by absolute and relative values for the responses obtained, from the sum of all factors.

## Results

To facilitate understanding, the results were separated into 3 categories for display: 1. Website, 2. Evaluation of the website, and 3.Profile of the evaluators.

1. Website

**Fig. 1:**
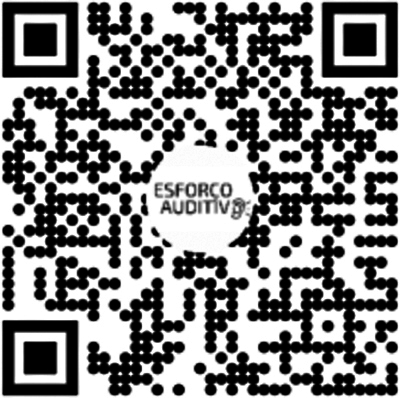
QR code to access the website.

The website has 10 tabs, namely: Home, Listening Effort, Understanding the Cognitive Process, Objective Assessment, Subjective Assessment, Behavioral Assessment, Therapeutic Resources, Testimonials, References and Contact, which can be accessed at https://esforco-auditivo.webnode.com or by the QR code available in Figure 1 , below:

For the elaboration of the website, black, red, and white colors were preferably used, alternating between background color, font color and emphasis color. The choice of large font, for ease of viewing, toggled between bolded and regular, to make certain words stand out. The italics feature was used only in foreign words.

To compose the site, complementary materials were prepared and adapted in order to facilitate understanding of the topic. Free translations of three images were performed, as follows:

- The Framework for Understanding Effortful Listening (FUEL); 10
- The original model proposed by Kahneman in 1973 11 to explain the listening effort;
- The original article's image demonstrating factors related to the listener, environment or interlocutor that can influence the interpretation of the auditory message. 12

To facilitate the understanding of FUEL, a video was created explaining the steps that make up the model. A video demonstrating the application of the dual task paradigm was also prepared.

2. Website Evaluation

The evaluation of the website was carried out through the application of the SAM questionnaire via an online form. As a result, 70 responses were obtained. Only one response was excluded from the sample because the evaluator indicated that he had already answered the questionnaire, and other 5 was excluded because the person indicated that were a student. In total, 64 responses were considered, making it possible to obtain absolute values of the average for each question.

As for the “Content” of the website, the evaluators had to answer four questions, considering: if the purpose was evident, if the content was about behavior, if the content was focused on its purpose, and if the content highlighted the main points.

Regarding the items corresponding to the “Literacy Requirement”, the evaluators were asked about the reading level; more specifically, if the material uses the active voice, if it uses vocabulary with common words, if the context comes before new information, and whether learning is facilitated by topics.

The third item, referring to “Illustrations”, presents questions regarding: if the purpose of the illustration referring to the text is clear, if the types of illustrations, if the figures/illustrations are relevant, if the lists, tables, and other visual items have an explanation and if the illustrations have a caption.

To evaluate the questions related to “Layout and Presentation”, the evaluators were asked about: adequacy of layout characteristics, font size and type, and whether subtitles were used.

Regarding the item “Stimulation and Motivation for Learning” generated by the website, the evaluators should answer the questions if: the website uses interaction, if the guidelines are specific and provide examples, and if the website stimulates motivation and self-efficacy.

As for “Cultural Adequacy”, the participants were asked if the content presented: similarity to their logic, language and experience and regarding the adequacy of the cultural image and examples. Respectively, the following average results were obtained, shown in Table 1 .

**Table 1:** Analysis of absolute values regarding the averages of the evaluator's responses through the SAM for evaluation of health related information for adults (n = 64)

Question	Answers (%)		
Content	Great	Adequate	Inadequate
1a	50.0	48.43	1.57
1b	35.93	62.53	1.57
1c	54.68	43.75	1.57
1d	56.25	42.18	1.57
**Literacy Requirement**			
2a	54.68	40.62	4.68
2b	37.5	59.37	3.12
2c	50.0	48.43	1.57
2d	53.12	39.06	7.81
2e	39.06	57.81	3.12
**Graphics**			
3a	56.25	40.62	3.12
3b	51.56	43.75	4.68
3c	57.81	40.62	1.57
3d	45.31	54.68	–
3e	45.31	48.43	6.25
**Layout and Type**			
4a	43.75	50.0	4.68
4b	50.0	46.87	3.12
4c	46.81	51.56	1.57
**Learning Stimulation & Motivation**			
5a	37.5	57.81	4.68
5b	57.81	40.62	1.57
5c	53.12	45.31	1.57
**Cultural Appropriateness**			
6a	45.31	40.62	14.06
6b	50.0	35.93	14.06

**Abbreviations:** SAM, suitability assessment of materials.

Analysis of absolute values regarding the averages of the evaluator's responses through the Suitability Assessment of Materials (SAM) for evaluation of health related information for adults (n = 64) ( Table 1 ).

In the average of all answers, a percentage of 47.76 answers considered the material as excellent, with 39.17 considering it adequate, and 13.05% as inadequate, with a total of 86.93 of the material considered adequate.

**Fig. 2:**
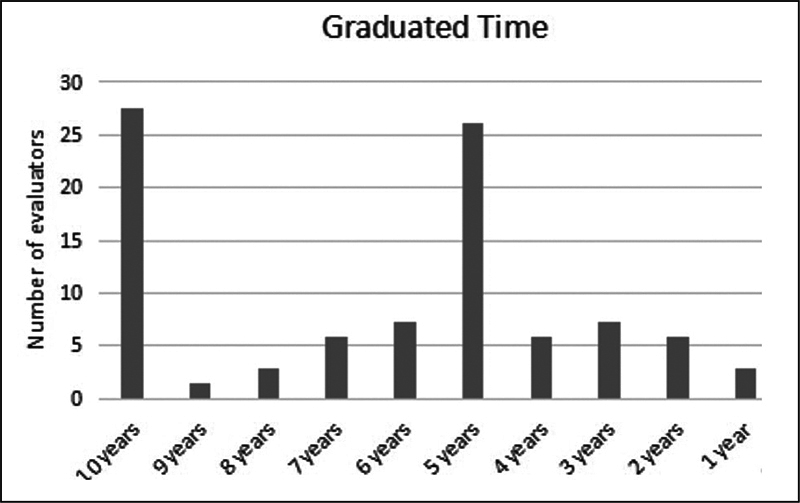
Graph 1–graduated time of evaluators.

**Fig. 3:**
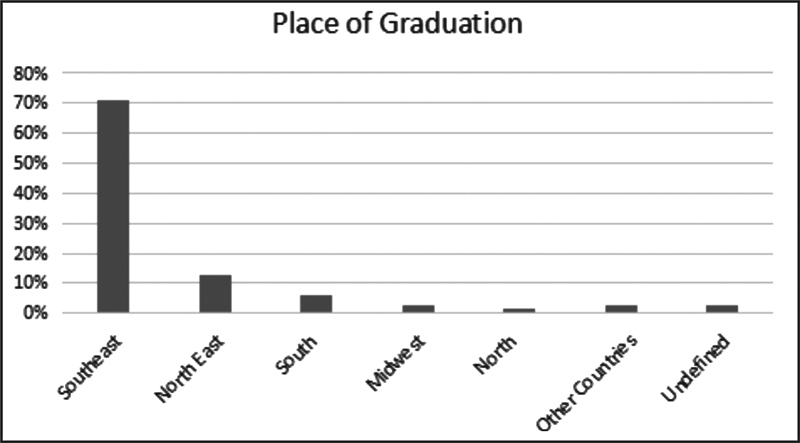
Graph 2–graduation place of evaluators.

3. Evaluators Profile:

Upon request in the form, the evaluators shared their time of graduation, as shown in graph 1, Figure 2 :

All evaluators were speech therapists and audiologists, and 79.68% reported experience in the area of Audiology. It was also possible to observe that the form reached all regions of the country, as well as two Colombian evaluators, appearing as “other countries” which is shown in graph 2 ( Figure 3 ). There were also people who did not clearly inform their location, and who appear in the graph as “undefined”.

## Discussion

As explained by Serrasqueiro, 13 it is convenient for educational materials to be prepared in an accessible way to all readers, including those with learning disorders. In this sense, we aimed to make the website's elements sequenced and correctly distributed on the page, with clear separation between elements, legible letters, and use of contrasts, as well as updated references to technologies that are frequently modified, representation of images and illustrations elaborated or adapted by the authors, and use of motivating elements, such as explanatory videos.

The elaborated material was evaluated by speech-language therapists and audiologists who verified the adequacy of the questions: content, literacy requirement, illustrations, layout, presentation, stimulation/motivation of learning, and cultural adequacy through SAM. Using the same instrument, another study evaluated written materials designed to provide information in a hospital. Among the conclusions, the researchers verified the importance of using tools to evaluate written educational materials, citing that, during their preparation, issues addressed in each item can be changed if they are inadequate. Therefore, each answer must be evaluated individually and critically, as some of them may be subjective. Thus, it is important for data analysis to be carried out in a psychometric manner by evaluators who are familiar with the subject. 14

One study also suggests the use of SAM for the evaluation of educational materials, emphasizing the importance of educators and creators adapting their didactic material to the target audience, carrying out a pilot study and using content validation checklists. 15 Such suggestions agree with the methodology used in the present study.

Considering that the website “Esforço Auditivo” was developed for professionals in speech-language pathology and audiology, it was recommended that the content was approached from basic concepts about listening effort to therapeutic resources available in the literature. Among the ten tabs available on the site, it was also discussed how the effort happens at the cortical level, intrinsic or extrinsic aspects to the individuals who present with listening effort, different populations that present with listening effort, several objective, subjective, and behavioral evaluation methods, and therapeutic resources.

As for the SAM item referring to the website's content, 49% of the evaluators considered the evidence of the website's purpose to be adequate, and 62.31% considered that the content adequately addressed behavior. When asked if the content was focused and if it highlighted the main points, respectively, 55.07% and 56.52% of the evaluators answered as excellent, as can be seen in Appendix 2 .

**Appendix 2:** QR Code to access the complete data table

Among the graduated evaluators, 76.81% reported having experience in the area of Audiology. This data supports the material's reliability, considering that, when evaluating an educational material, the participation of experts is essential, in order to avoid wrong assumptions and the risk of bias. 16

Analyzing the answers to each question of the literacy requirements item, shown in Table 1 , it can be observed that they were all considered as “adequate” or “excellent” by at least 90% of the evaluators. The language chosen to communicate with the target audience was based on technical terms in order to encourage professionals to keep reading. 17

Regarding the illustrations item, the use of real images instead of drawings for graphic representations was also planned considering the target audience, as proposed by other authors. 15 Furthermore, approval as “excellent” or “adequate” occurred in at least 94% of the responses for all aspects questioned. It is noteworthy that question 3d, which asks whether lists, tables, and other visual items contain explanations, was not marked as inappropriate by any of the evaluators.

According to what is proposed by the “Conceptual Framework for the Audiovisual Designer” 18 , the adapted images and the video prepared to explain FUEL were developed considering aspects of pedagogical demand, which define the content, educational objectives, and target audience along with the designer of the Institution's Educational Technology sector, as well as aspects of idealization, formatting, production, and validation of materials.

As for the layout, 46.37% of the evaluators considered the layout “excellent”. Of the 69 evaluators, 50.72% considered the font size and type to be optimal and the subtitles adequate, in view of what is shown in the results ( Table 1 ). Such findings coincide with a study on website layouts, which reports the need for a simple and well-defined structure that can be used easily. 19

The level of stimulation and motivation for learning is also assessed by the SAM, and the results obtained are shown in Table 1 . A study described the importance of motivation in the learning process. The author states that without motivation there is no learning, because it determines the level of effort that the student will employ in a given task, making it vital that the teacher is in constant search for strategies that provide stimulation and motivation. 20

Over 95% of the website evaluators considered that all the questions in the stimulation and motivation for learning item were excellent or adequate. Most evaluators considered that the website provides motivation and self-efficacy, that it uses interaction appropriately, and that the guidelines are specific and give examples.

Among the answers obtained, it was possible to observe that all the questions of the first five items evaluated had an average of “not adequate” answers of less than 8%, as shown in Table 1 . However, the item “cultural adequacy” had the lowest positive evaluation, with 14% of inadequacy for the question regarding the cultural images and examples. Regarding the questioning on whether the content was adequate to their logic, language, and experience, 13.04% considered it was not.

However, the result was satisfactory, considering the of the evaluators' responses for all items in the six categories, with approval of 96.24%, classifying the material as “superior.” 8 Therefore, according to the quantitative analysis of the answers, obtained by the averages of the absolute values, no modifications were necessary in the original project.

As for evaluators' profile, it was observed that most evaluators have graduated ten years ago or more. This data can be associated with this population's greater interest on the theme, aiming to stay updated. Another study that developed materials for permanent distance education observed in its results that the training time can influence its evaluation when the format is different than usual for that population. However, in the present study, no such relationship was observed (please see Appendix 2 ). 21

As for the technological aspect, we observed that in the weeks in which the evaluation form was available, it was answered by professionals from the five regions of Brazil, as well as by professionals from another country. This fact may reflect the ease of disseminating knowledge online, free of charge and easily accessible, as proposed. In 2021, Dos Santos Carvalho et al. 22 claimed that information technologies are essential in the propagation of knowledge at high speed, regardless of spatial barriers, allowing access to the most varied types of materials, in addition to providing the stimulation of several senses, simultaneously.

A recent study also discusses the importance of technology and continuing education, considering the scenario during the COVID-19 pandemic. The author highlights that there is a growing need to adhere to the changes of society that are increasingly digitally versed and, at this moment, technological strategies are essential for maintaining the learning processes at all educational levels. 23

## Conclusion

As proposed, it was possible to create the website “Esforço Auditivo”. The quality of the content, literacy requirements, illustrations used, layout, level of stimulation, and learning motivation generated by the site, in addition to its cultural adequacy, were evaluated by speech therapists from all over the country. After obtaining the website's “superior” classification, it was considered ready to be disseminated through social networks, then made available in the public domain in an online format and free of charge for professionals.

It is noteworthy that a longitudinal work will be carried out with the content covered, updating, and translating the original material into other languages by students from the proposing institution.

In conclusion, the website was evaluated and validated to be made available online.
